# Association between tuberculosis in men and social network structure in Kampala, Uganda

**DOI:** 10.1186/s12879-021-06475-z

**Published:** 2021-09-30

**Authors:** Paige B. Miller, Sarah Zalwango, Ronald Galiwango, Robert Kakaire, Juliet Sekandi, Lauren Steinbaum, John M. Drake, Christopher C. Whalen, Noah Kiwanuka

**Affiliations:** 1 Odum School of Ecology, University of Georgia, Athens, GA 30602 USA; 2 Kampala Capital City Authority, Kampala, Uganda; 3 Makerere University School of Public Health, Kampala, Uganda; 4 Global Health Institute, College of Public Health, University of Georgia, 100 Foster Drive, Athens, GA 30602 USA; 5 Department of Epidemiology and Biostatistics, College of Public Health, University of Georgia, Athens, GA 30602 USA

**Keywords:** Tuberculosis, Social network, Male-bias, Contact patterns

## Abstract

**Background:**

Globally, tuberculosis disease (TB) is more common among males than females. Recent research proposes that differences in social mixing by sex could alter infection patterns in TB. We examine evidence for two mechanisms by which social-mixing could increase men’s contact rates with TB cases. First, men could be positioned in social networks such that they contact more people or social groups. Second, preferential mixing by sex could prime men to have more exposure to TB cases.

**Methods:**

We compared the networks of male and female TB cases and healthy matched controls living in Kampala, Uganda. Specifically, we estimated their positions in social networks (network distance to TB cases, degree, betweenness, and closeness) and assortativity patterns (mixing with adult men, women, and children inside and outside the household).

**Results:**

The observed network consisted of 11,840 individuals. There were few differences in estimates of node position by sex. We found distinct mixing patterns by sex and TB disease status including that TB cases have proportionally more adult male contacts and fewer contacts with children.

**Conclusions:**

This analysis used a network approach to study how social mixing patterns are associated with TB disease. Understanding these mechanisms may have implications for designing targeted intervention strategies in high-burden populations.

## Background

Although tuberculosis (TB) is both a treatable and preventable disease, it remains one of the leading causes of death worldwide. Each year an estimated 10 million people fall ill and 1 million people die of TB [1]. In addition, approximately 25% of the world’s population has a latent infection with *M. tuberculosis (Mtb)* and is at risk of progressing to TB disease [2]. Notification of TB disease is more common in males than in females with an average of 1.8 cases notified among men for each woman globally in 2017 [1]. One explanation for the excess of cases among men is that they have greater access to health care than women. Although this may contribute to disparity among men and women in some places, TB prevalence surveys, which control for access to care, also find male-bias in prevalent TB [3, 4]. Understanding how and why the burden of TB differs by sex may be contribute to finding and treating undetected TB cases in the community.

As explanations for male-bias in TB (i.e., excess TB cases among men), many propose that men have greater susceptibility to infection or more frequent opportunities for exposure [5, 6]. A number of factors have been put forward as mechanisms for heightened susceptibility in men. In most countries, men smoke more cigarettes than women, and per capita smoking rates explain roughly one-third of the variation in country-level male-bias in case reports [7], perhaps due to toxic lung injury and reduced immune cell function [8] leaving them more susceptible to infection. Alcohol use is also identified as a risk factor for TB disease as it may have immunosuppressive effects [9]. These behavioral factors and other hormonal and physiological factors likely play a role in determining sex-specific susceptibility to Mtb [5, 6], though we do not know the combined extent to which they explain male-bias across populations nor do we know the full spectrum of possible mechanisms.

Apart from susceptibility, men may also be exposed to undetected, infectious TB cases more often than women. This unknown exposure may be determined in part by the social role men fill and how social roles influence mixing with others in their community [10]. For instance, in Uganda adult men travel more often than women while more than one-quarter of adult women identify as housewives [11], potentially causing higher exposure rates among men due to the number of contacts or centrality of men within social networks. Alternatively, male-bias could be perpetuated because men preferentially interact with men who are more likely to be infected than women [10]. Sex assortativity could further magnify spread among men if males were more likely to transmit infection to their close contacts than females, as some studies have found [12–14]. Compared with biological differences in susceptibility, few studies have directly examined whether differences in network centrality and mixing patterns by sex, driven by gender roles and potentially causing changes to exposure rates to infectious cases, can amplify tuberculosis burden among men.

One way to understand whether sex differences in network position or mixing patterns affect a person’s likelihood of developing TB disease is to compare the social networks of TB cases with networks of healthy controls in an area endemic for TB. To this end, we analyzed the structure of a large social network in Kampala, Uganda centered on male and female index participants who were either recently diagnosed with active TB cases or were community-matched controls who were asymptomatic [15]. We used the social network data to test whether network position and mixing patterns within networks were associated with TB. For network position, we predicted that (1) greater network centrality would be associated with TB disease [16, 17] and (2) that men would be more central in their social networks. For mixing patterns, we predicted that (1) there would be preferential mixing by sex and (2) that TB cases would have more contact with men than controls.

## Methods

### Data collection

This study took place from 2013 to 2017 in the Rubaga Division of Kampala, Uganda. Rubaga is an urban area where approximately 300,000 people reside. The prevalence of TB in Uganda is one of the highest in the world [1] and, in Rubaga Division, nearly one half of the population may be latently infected [18]. This area’s urban landscape, high male:female case notification rate (2.4:1) [1], and high prevalence of infection make it a relevant place to study the factors affecting TB spread in endemic populations.

To characterize the social networks of people living in Rubaga, we delineated the egocentric networks of TB cases and frequency-matched, community controls without symptoms for TB from that urban suburb. The methods used in this study have been previously described in [15]. Briefly, we enrolled adult (15 years or older) TB index cases (n = 123) from the National Tuberculosis and Leprosy Control Programme who presented with their first episode of microbiologically-confirmed (i.e., Xpert MTB/RIF or sputum microscopy) pulmonary TB. Index controls (n = 124) were frequency-matched to index cases according to age-group, sex, and parish and recruited through door-to-door surveys. Active TB was excluded in index controls if there were no signs or symptoms of the disease. Controls were counseled to report any symptoms of TB disease, but none did during the study. For index participants, we collected information on demographic characteristics including age and relationship status (single, divorced, widowed, monogamous, polygamous); relationship was re-categorized as single (single, divorced, widowed) or in a relationship (monogamous, polygamous). We used two-way ANOVAs to compare age and a Chi-square test to compare relationship status by index type (cases or control) and sex.

**Fig. 1:**
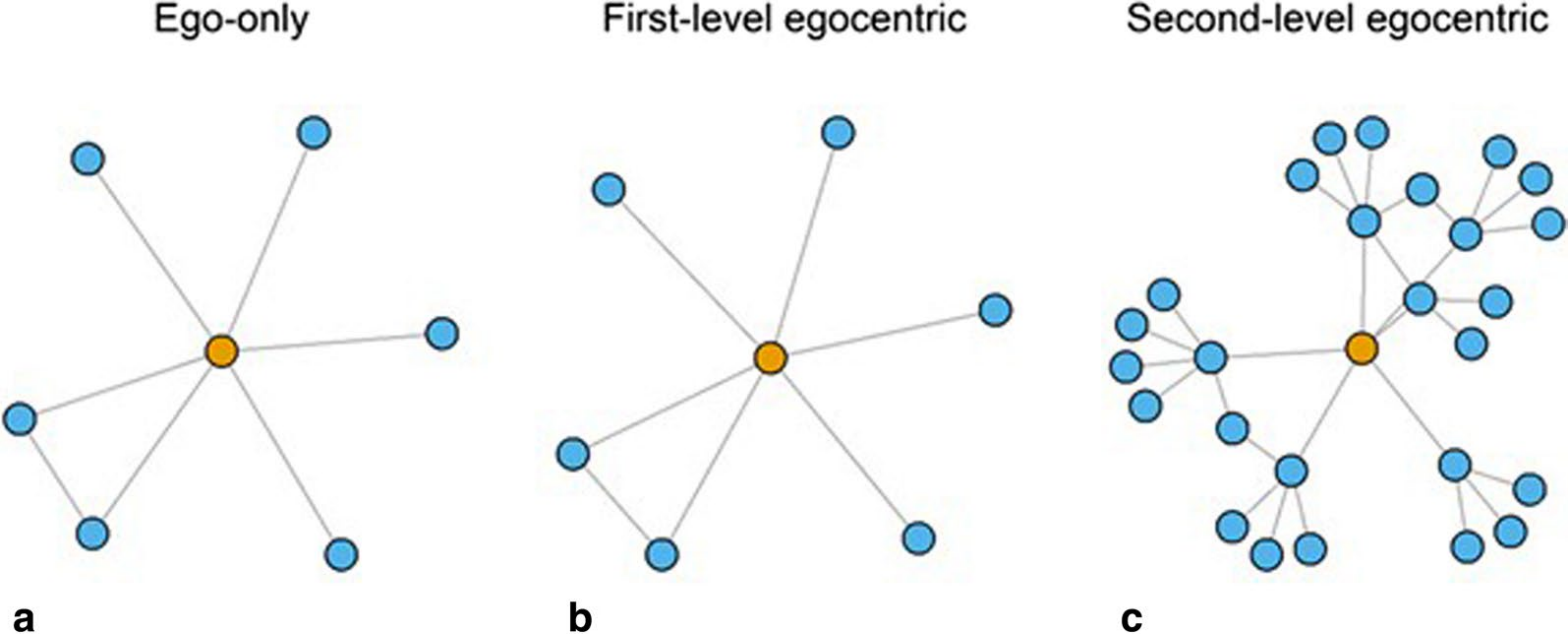
Types of egocentric network sampling varies in the amount of information collected outward from the index individual. Index individuals, shown in yellow, list their contacts in ego-only sampling (**a**). In first-level egocentric sampling (**b**), index individuals additionally indicate whether their contacts are also contacts. In second-level egocentric sampling (**c**), contacts of index individuals list their own contacts. The social network in the Kampala study utilized a second-level egocentric design. We used sensitivity analyses to understand how these types of egocentric sampling designs alter estimates of network centrality statistics

Following recruitment of index participants, trained interviewers ascertained their social networks in a two-step process. In the first step, index participants listed members of their households and all individuals living outside their household with whom they had a personal relationship or regular close contact. Close contact was defined as being within talking distance for more than 4 h on more than one occasion. In the second step, each of these first-level contacts were asked to list their contacts using the exact same approach. Unless there was a suspicion of active TB, field nurses did not trace the second-degree contacts. This sampling methodology was an extended form of egocentric sampling, which we will refer to as “second-level egocentric sampling” in the remaining sections. Second-level egocentric sampling differs from classic egocentric network sampling in the additional layer of contacts collected (Fig. 1).

Social network forms (Additional file 1) completed by trained interviewers, reviewed at point-of-contact and prior to data entry with optical scanning software (TeleForms). All data were read and verified by a data clerk; item analysis detected incomplete or out-of-range entries which were corrected with reference to source documents before proceeding Participants were assigned unique identifiers; we screened for duplicated individuals appearing in multiple networks with a software program designed to find similar rows of data (dedupe.io). All duplicates that were identified were hand-checked by co-authors R. Kakaire and R. Galiwango.

### Social network analysis

We assumed social networks were undirected for the purposes of this analysis. We described the large-scale features of the social network including its size (number of members), component distribution (number of sub-networks connected to each other through common contacts), and mean degree (average number of contacts per individuals). Additionally, we compared the degree distribution of index participants, first-level, and second-level contacts and used a two-way ANOVA to compare the degree distribution of index participants and that of first-level contacts. We compared estimates of node position among index participants by index type (case or control) and sex. Centrality statistics included node degree, betweenness [19], and closeness [20] (definitions in Table 1). To determine whether men were more clustered with TB cases than women, we calculated the network distance to a TB case. We used two-way ANOVAs to determine whether dependent variables were associated with independent variables sex (male or female) and index type (TB case or control).

**Table 1:** Social Network Estimates used to describe the Node Position of Index Individuals ($s\in \{\mathrm{1,2},..n\}$) within the Kampala Network

Statistic	Definition	Equation	Notation
Node degree, ${k}_{s \in \mathrm{1,2},\dots n}$	Number of adjacent edges	$\sum\nolimits_{j = 1}^{N} {A_{s,j} }$	Adjacency matrix, ${A}_{ij}=1$, if we identified contact between $i,j$
Betweenness, ${b}_{s\in \mathrm{1,2},...n}$	Number of times node is on shortest path between pairs of other nodes^a^	$\sum\nolimits_{u \ne s \ne v} {\frac{{\sigma_{uv} \left( s \right)}}{{\sigma_{uv} }}}$	${\sigma }_{uv}$ is the total number of shortest paths from node $u$ to $v$ and ${\sigma }_{uv}\left(s\right)$ is the number of those paths that pass through $s$
Closeness, ${c}_{s\in \mathrm{1,2},...n}$	Inverse of the average length of shortest path to all other nodes^a^	$\frac{1}{{\sum\nolimits_{i \ne s} {d_{si} } }}$	${d}_{si}$ is the network distance between nodes $s$ and $i$
Distance to TB case, ${y}_{s\in \mathrm{1,2},...n}$	Network distance to a TB case^a^	${\text{min}}\left( {d_{st, t \ne s} } \right)$	$t$ is the set of TB cases

^a^Network distance, closeness, and betweenness were calculated within the giant component because path length is not defined for disconnected graphs

To assess patterns of mixing by sex and age in the network, we compared the proportion of contacts occurring within-sex, between-sex, and with children (< 15 years old) in the network for index participants. We also quantified sex-assortative mixing with the assortativity coefficient, $r$ [21]. These coefficients are based on the matrix, ${E}_{ij}$, describing the fraction of all edges that connect a node of type $i$ to type $j$, such that the diagonal ${E}_{ii}$ represents within-group edges, the off-diagonal represents between-group edges, and $\sum_{ij}{E}_{ij}=1$. If ${a}_{i}=\sum_{j}{E}_{ij}$ and the network is undirected, the assortativity coefficient is defined as $r=\frac{\sum_{{\varvec{i}}}{E}_{ii}-\sum_{{\varvec{i}}}{a}_{i}^{2}}{1-\sum_{{\varvec{i}}}{a}_{i}^{2}}$**.** Assortativity coefficients range from − 1 to 1, with larger, positive values corresponding to assortative networks and negative values being more disassortative (coefficients close to 0 indicate no assortativity for that variable) [21].

### Sensitivity analyses

We examined robustness of network centrality statistics to egocentric sampling. First, we simulated small-world [22] and scale-free [23] networks across a range of networks sizes 50 times. Then we subjected each network to three forms of egocentric sampling [24], including the one used in this study (Fig. 1, Additional file 2). To understand how sampled network statistics related to underlying network statistics of index participants, we calculated correlation coefficients. To understand how social networks in Kampala related to small-world and scale-free networks, we calculated the clustering coefficient (i.e., the probability that neighbors of a node are also connected [25]) and fit to a power-law degree distribution [26] because small-world networks are characterized by high clustering coefficients and scale-free networks by a power-law degree distributions [27]. We performed a second sensitivity analysis focusing on estimates of assortativity (Additional file 2). We again calculated the correlation between sampled assortativity and true assortativity of the underlying network to assess robustness to egocentric sampling.

All network analyses and simulations were completed in R (4.0.0) using the package igraph [28]. R scripts for sensitivity analyses are available on https://github.com/DrakeLab/miller-tb-centrality.

#### Ethics considerations

The study was approved by the University of Georgia Institutional Review Board, the Higher Degrees Research and Ethics Committee at Makerere University School of Public Health, and approved by the Uganda National Council for Science and Technology. Written informed consent was obtained for all adult participants (18 years and older); for participants aged 12–17, written consent was obtained by the participant’s parent or guardian and written assent from the participant; for minors (< 12 years), written consent was obtained by the participant’s parent or guardian.

## Results

### Overall network structure

Index participants (n = 123 cases, n = 124 controls) listed 2418 contacts (first-level contacts) of which 1930 agreed to enroll in the study and subsequently identified 9175 second-level members. Index participants identified on average two more contacts than first-level participants (10.4 vs. 8.2) (Mann–Whitney U-test, p < 0.0001). Thus, 2177 members (index participants and enrolled first-level participants) reported 14,307 edges.

The resulting network of 11,840 members of whom 6507 were males, 5333 females, 9720 adults (at least 15 years old), and 2002 children less than 15 years old (age was not identified for 118 members). Overall degree, including second-level contacts, was 2.4 (± 0.03, SE). Despite low connectivity of second-level contacts, all 247 index networks were distributed in one of 47 network components after joining networks with common contacts (Additional file 2: Fig. S1). One component connected 9,974 (84%) members and 187 (75%) index participants (102 controls and 85 cases).

### Demographic characteristics of index participants

As described in [15], of the 247 index participants, 169 were men and 78 were women. Controls were almost twice as likely to be in a relationship than TB cases (${\chi }^{2}$=13.9, df = 1, P = 0.0002) (Table 2). Male index participants were six years older than female index participants on average (F_1, 246_ = 26.8, P = 4.68 × 10^−7^) (Table 3).

**Table 2:** Demographics and Social Network Estimates for Index Individuals stratified by Index type. Values indicate the number of individuals (proportion) or mean ($\pm$ standard errors) for each variable

	Case n = 123	Control n = 124	Sig
Age	30.6 (± 0.90)	32.0 (± 0.85)	
Monogamous or polygamous relationship	40 (0.33)	70 (0.56)	**
Node position			
Degree	10.7 (± 0.36)	10.2 (± 0.36)	
Closeness	0.076 (± 0.001)	0.077 (± 0.001)	
Betweenness	0.009 (± 0.002)	0.02 (± 0.002)	**
Distance to case	3.2 (± 0.2)	3.5 (± 0.2)	
Mixing variables			
Proportion of all contacts with adult men	$0.47 (\pm 0.02)$	$0.36 (\pm 0.03)$	**
Proportion of all contacts with adult women	$0.37 (\pm 0.02)$	$0.40 (\pm 0.02)$	
^a^Proportion of all contacts with children	$0.16 (\pm 0.02)$	$0.23 (\pm 0.02)$	**
^b^Proportion of all contacts occurring within HH	$0.23 (\pm 0.02)$	$0.37 (\pm 0.03)$	**
Proportion of HH contacts occurring with children	0.28 $(\pm 0.02)$	0.32 $(\pm 0.02)$	

^a^Adults $\ge$ 15 years old, children $<$ 15

^b^HH: Household

$$**$$Significant difference (p < 0.05) between means by index type (case, control)

**Table 3:** Demographics and Social Network Estimates for Index Individuals stratified by Index Type and Sex

	Female		Male		Sig
	Case n = 39	Control n = 39	Case n = 84	Control n = 85	
Age	$25.6 (\pm 0.89)$	$28.0 (\pm 1.11)$	$32.9 (\pm 1.16)$	33.8 $(\pm 1.08)$	*
Monogamous or polygamous relationship	12 (0.31)	22 (0.56)	28 (0.33)	48 (0.57)	**
Node position					
Degree	$10.4 (\pm 0.70)$	$9.95 (\pm 0.4)$	$10.8 (\pm 0.4)$	$10.3 (\pm 0.5)$	
Closeness	0.077 $(\pm 0.002)$	$0.076 (\pm 0.003)$	0.075 $(\pm 0.002)$	$0.078 (\pm 0.002)$	
Betweenness	0.005 ($\pm 0.001)$	$0.015 (\pm 0.003)$	$0.010 (\pm 0.002)$	$0.022 (\pm 0.003)$	**
Distance to case	$3.4 (\pm 0.3)$	$3.8 (\pm 0.3)$	$3.0 (\pm 0.2)$	$3.3 (\pm 0.2)$	
Mixing variables					
Proportion of all contacts with adult men	$0.34 (\pm 0.04)$	$0.22 (\pm 0.02)$	$0.53 (\pm 0.03)$	$0.43 (\pm 0.03)$	*,**
Proportion of all contacts with adult women	$0.41 (\pm 0.04)$	$0.44 (\pm 0.03)$	$0.35 (\pm 0.02)$	$0.38 (\pm 0.02)$	*
^a^Proportion of all contacts with children	$0.26 (\pm 0.03)$	$0.34 (\pm 0.03)$	$0.12 (\pm 0.02)$	$0.19 (\pm 0.02)$	*,**
^b^Proportion of all contacts occurring within HH	$0.31 (\pm 0.04)$	0.45 $(\pm 0.04)$	0.19 $\left(\pm 0.02\right)$	$0.33 (\pm 0.03)$	*,**
Proportion of HH contacts occurring with children	0.32 $(\pm 0.04)$	0.39 $(\pm 0.03)$	0.25 $(\pm 0.02)$	0.29 $(\pm 0.02)$	*

Values indicate the number of individuals (proportion) or mean ($\pm$ standard errors) for each variable

^a^Adults $\ge$ 15 years old, children $<$ 15

^b^HH: Household

*Significant difference (p < 0.05) between means by index sex (male, female)

**Significant difference (p < 0.05) between means by index type (case, control)

### Node position of index participants

**Fig. 2:**
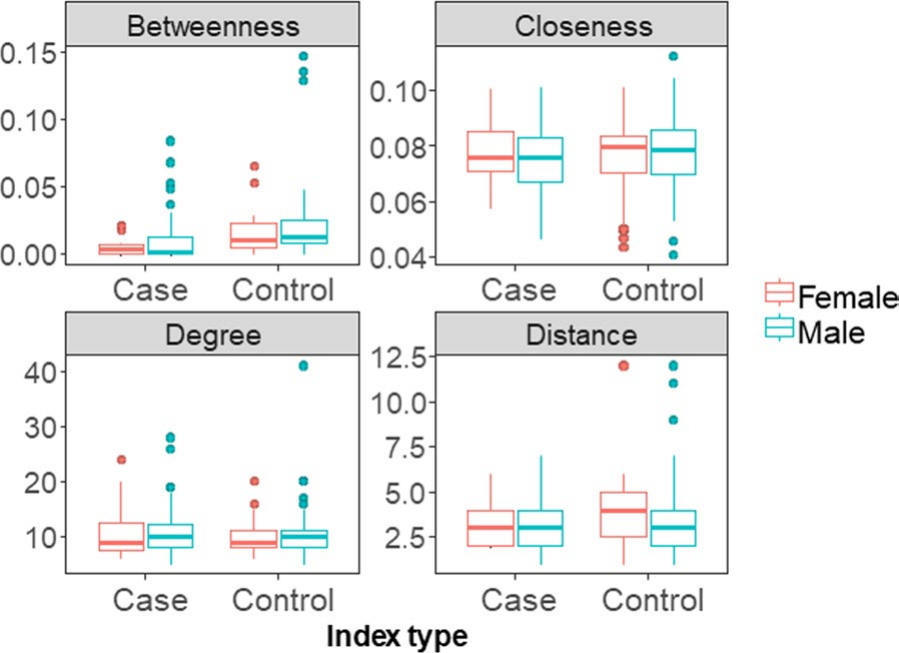
There were few differences in node position (degree, betweenness, closeness, and network distance to index cases) by index type and sex. Closeness, betweenness, and network distance to index cases were calculated for index individuals in the giant component (i.e., the largest connected network component). Boxplots show interquartile regions and outliers

In our analyses of node position, there was little variation among index participants stratified by type (Table 2) and sex (Fig. 2, Table 3, Additional file 2: Fig. S2). The mean degree (number of contacts) of index cases and controls were 10.7 and 10.2, respectively, but this difference was not statistically significant (Table 2). Other estimates of node position (betweenness, closeness, and network distance to TB cases) were measured for the 187 index participants in the giant component. Index men tended to have shorter distances to TB cases than women, but not significantly so (${\mathrm{F}}_{\mathrm{1,183}}$ = 2.78, P = 0.096). Only network betweenness differed between cases and controls, and controls had higher betweenness than cases (${\mathrm{F}}_{\mathrm{1,183}}$ = 12.73, P = 0.0005). Closeness of index participants was not higher among men or among TB cases. Since a significantly higher proportion of controls were in monogamous or polygamous relationships, we stratified network position statistics by relationship status (Additional file 2: Table S1). There was no difference in network position (degree, betweenness, closeness, or distance) between index participants that were single and those in a relationship.

### Social mixing patterns of index participants

**Fig. 3:**
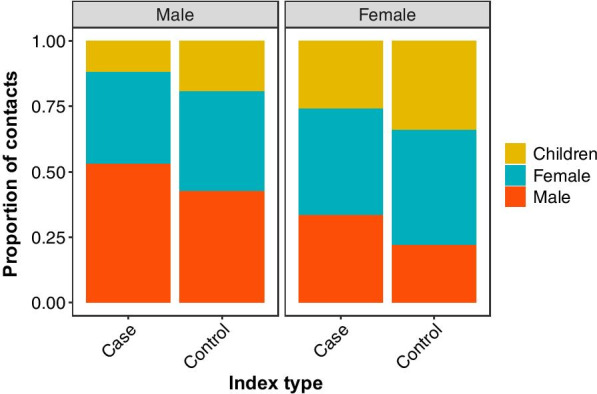
Proportion of all contacts of index individuals that are with adults and children (< 15 years old) reflects high levels of assortative mixing in this population. There were more within-sex edges than between-sex edges. There were also more edges between adult females and children than adult males and children

The distribution of contacts reported by index participants indicated strong evidence of preferential mixing by sex and higher levels of contact between women and children (< 15 years old) than men and children (Fig. 3, Tables 2, 3). Across the observed network, same-sex edges were almost twice as common as between-sex edges (9079 vs. 5228), with a sex-assortativity coefficient of 0.26 ($\pm 0.01,$ SE). Of the contacts reported by index participants, the proportion that were adult men varied significantly by index sex (F_1, 246_ = 37.1, P = 4.23 × 10^−9^) and type (F_1,246_ = 12.3, P = 0.0006) with TB cases and men having a higher proportion of their reported contacts with other men. The proportion of contacts with adult women varied significantly by index sex (F_1, 246_ = 4.213, P = 0.04) but not index type. Overall, index women reported approximately two times more contact with children than index men (0.30 and 0.15, respectively) and this was a significant difference (F_1, 246_ = 36.5, P = 5.67 × 10^−9^). In addition, index controls reported a slightly higher proportion of contacts with children than TB cases (0.33 vs. 0.26; F_1,246_ = 11.4, P = 0.0008). Relationship status was not associated with any mixing variables (Additional file 2: Table 1).

### Sensitivity analyses

**Fig. 4:**
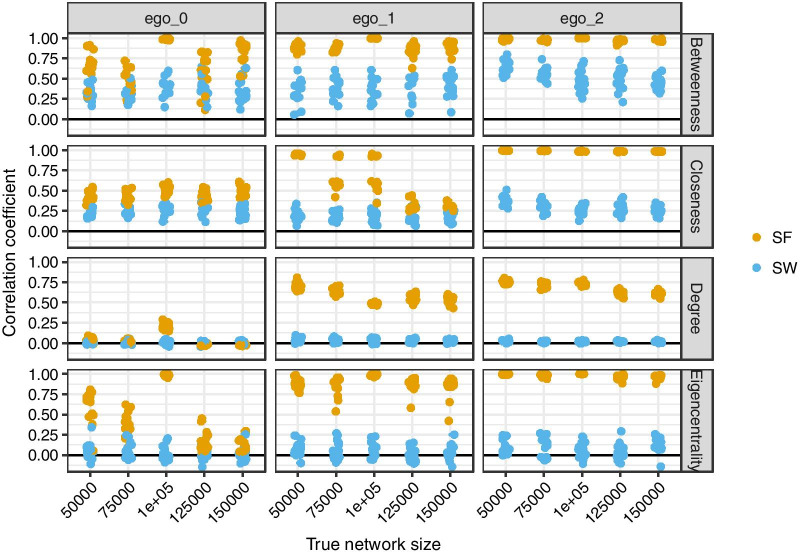
Sensitivity analyses showing the correlation between true node centrality and estimated node centrality depends on the underlying network type being sampled and the type of egocentric sampling used. We simulated 15 replicates of each network type (scale-free and small-world) across a range of network sizes $\left(5 \times {10}^{4},7.5\times {10}^{4},1\times {10}^{5},1.25\times {10}^{5},1.5\times {10}^{5}\right)$ all with mean node degree of 10. We then simulated the process of three types of egocentric sampling (ego_0, ego_1, and ego_2) and calculated the correlation of estimated centrality with true centrality. The black line indicates no correlation between sampled node statistics and true node statistics and the red triangle shows the mean across all replicates. Since we assumed perfect recall, we calculated the correlation in sampled degree on all nodes in the sampled network (i.e., not just the ego). Betweenness of egos was estimated from simulated networks by capping the number of search algorithm of shortest paths to 25

Overall, estimates of node centrality statistics were differentially impacted by network type, sampling type, and underlying network size (Fig. 4). When the underlying network being sampled was a scale-free type, all centrality estimates from egocentric samples were correlated with the true centrality in second-level samples $\left(\rho >0.4\right),$ indicating identifiability of node position statistics compared in this analysis. In contrast, node position was less identifiable when sampling from small-world networks. The Rubaga social network we analyzed had a low clustering coefficient and a degree distribution consistent with either a power-law (i.e., matching that of scale-free networks) or log-normal distribution (i.e., sum of multiple normal-distributions), but we could not distinguish between these two distributions (Additional file 2: Figs. S3 and S4). In general, second-level egocentric sampling was superior to ego-only and first-level egocentric sampling. Estimates of node position from egocentric samples were not highly sensitive to the range of network sizes chosen to represent a sub-population of the Rubaga Division. In a separate sensitivity analysis of network assortativity, estimated assortativity from egocentric networks was highly correlated ($\rho =0.999)$ with true assortativity of underlying networks (Additional file 2: Fig. S5).

## Discussion

Social contact networks alter infection patterns of infectious diseases [29–31] and, for TB specifically, social network analysis helps explain clusters of transmission in outbreaks among high-risk individuals [32, 33]. Our study extends the previous analyses in non-endemic areas to examine the structure of a large social network in Kampala, Uganda, where TB is endemic. Our primary interest was to understand how social network structure affected the pattern of TB in the community and, in particular, whether network structure could explain the male-bias observed in TB. Based on mathematical models [16], we predicted that recently diagnosed TB cases would be more central in social networks than index controls. Further, we predicted that men would be more central in networks than women. Contrary to our predictions, we found few differences in node centrality between index cases and controls and between men and women. Based on known preferential mixing patterns [29, 34], we also expected to find preferential mixing patterns by sex and age. Indeed, we observed assortativity by both sex and age. The novel finding was the stark differences in mixing patterns between index cases and controls that could not be explained by age and sex. These findings suggest social network structure may perpetuate male-bias in TB by disproportionately leading men to have more contact with TB cases than women.

In this study population, the contact patterns of index cases and controls differed in a number of notable ways. First, index cases reported proportionally more male contacts than controls, even when accounting for sex, age, and relationship status of the index participant. Thus, not only did we find strong assortative mixing by sex among adults, but we also showed that having proportionally more male contacts is actually associated with being a TB case; that is, men may be positioned within their networks to be exposed more often to TB cases and thereby acquire new infection. This observation extends the idea that men are source cases to a disproportionate amount of new infections [10]. Second, men also had proportionally fewer contacts with children than index women, consistent with other studies of age- and sex-specific contact patterns [10, 34]. These variables were not related to age or relationship status of index participants. Last, index cases and men had a lower proportion of within-household contacts than controls or women. In other studies from Rubaga Division, investigators found that extra-household mixing was a risk factor for latent tuberculosis infection [18]; our observations also show how extra-household mixing may be associated with being a TB case. All together, these observations suggest that the sex-specific social mixing, which is consistently found among adults in many populations [34], may amplify exposure and consequent patterns of male-bias in TB prevalence and notification rates.

As for differences in network position, we found little variation between men and women and between index cases and controls. The only difference we did find was that index controls had slightly higher betweenness-centrality than index cases. This higher centrality may be because index controls were more likely to be in a relationship and perhaps better connected to their community. This point is further supported by more index controls than index cases in the largest network component. Still, these findings are surprising because models predict increased network centrality should increase infection likelihood [35]. Indeed, for latent infection, one study in Japan showed that among social contacts of TB cases those with higher betweenness-centrality had a higher likelihood of latent infection [17].

So why did we fail to see differences in network position by sex or index type in this study? Possibly, our sampling approach masked our ability to detect differences in network centrality (discussed below). Alternatively, we might have seen higher centrality among recently *infected* network members but we recruited sick (recently diagnosed) cases who may have been infected long ago due to the long and variable latent period of *M. tuberculosis*. Thus, the network position of index participants at the time of infection could be much different than it was at the time of enrollment in our study. Next, it is possible that we misclassified some index controls who had asymptomatic TB. Although possible, this type of misclassification was infrequent and not likely to bias the study results. Finally, in TB endemic settings, it is also possible that social networks of close contacts are not as strong a predictor of infection as more casual encounters [36]. Despite the negative findings about network position and participants recently diagnosed with TB, this analysis presents exciting new dimensions for the epidemiological and modeling communities about when more central network members are at greater risk for infection and how network-based approaches can best be used to find infected network members [35].

Our findings are based on partially-sampled social networks. To understand how sampling could change the response variables, we performed sensitivity analyses. Although our simulated networks were not subject to mechanisms of data incompleteness (e.g., imperfect recall) other than the sampling protocol itself (this has been analyzed elsewhere [37]) our sensitivity analyses aid in the interpretation of our findings. We found higher correlation of centrality estimates from egocentric samples with underlying networks in scale-free networks than in small-world networks. Since the Rubaga social network was only partially sampled, we cannot be certain whether the full, underlying network resembles a small-world or scale-free more closely but the observed degree distribution suggests a closer resemblance to scale-free graphs (Additional file 2: Fig. S4). Thus, we should have been able to distinguish highly central nodes from less central nodes in egocentric samples, but our findings about node position should be interpreted cautiously in light of our sensitivity analysis. Importantly, separate sensitivity analyses showed that assortativity statistics are robust to egocentric sampling. In summary, our sensitivity analyses allowed us to understand the impacts of network sampling on the response variables and we advocate simulation approaches to better understand real-world network data.

## Conclusions

The effects of gender-related social mixing patterns on the spread of *M. tuberculosis* are being increasingly recognized [34]. Although men were not more central in their social networks than women, there was a preferential and higher level of contact between recently diagnosed TB cases and other men within their networks, thereby providing novel insight into how social mixing patterns could drive male-bias in TB. Whether these observed levels of assortativity are pronounced enough to drive observed levels of male-bias is an open question for future research. For applied purposes, these findings could inform future intervention strategies such as prioritization of screening of men who have many male contacts.

## Supplementary Information

**Additional file 1.** Study data collection forms.

**Additional file 2.** Supplementary analysis.

## Data Availability

The datasets generated and analyzed during the current study are not publicly available due to participant confidentiality and vulnerability of the study population but are available from the corresponding author on reasonable request. The computer code used for this analysis is available in the GitHub code repository (https://github.com/DrakeLab/miller-tb-centrality).
