# Supplementary material for: Association between tuberculosis in men and social network structure in Kampala, Uganda
Source: BMC Infect Dis. 2021 Sep 30;21:1023. doi: 10.1186/s12879-021-06475-z (PMC8482622; doi:10.1186/s12879-021-06475-z)
Supplement: Supplementary file 1 — Additional file 1. Study data collection forms. [file 12879_2021_6475_MOESM1_ESM.pdf]

40782

MAKERERE UNIVERSITY- UNIVERSITY OF GEORGIA
COMMUNITY HEALTH AND SOCIAL NETWORKS OF TUBERCULOSIS
SOCIAL NETWORK FORM: TB CASE

Network ID ☐☐☐☐☐ Int Date ☐☐ / ☐☐ / 2 0 ☐☐ Contact serial# ☐☐

Explanation: You have given us the names of people you associate with (social contacts). We would like to ask you some questions about the nature of your relationship with those individuals one at a time.

1.What is the nature of your relationship with [Name] now?

☐☐

01= Spouse
02= Child
03= Sibling
04= Friend
05= Stranger
06= Co-worker
07= Student colleague
08= Relative
09= Acquaintance
88= Other (Specify) ☐

2.Is [Name] a new contact to you?(Became a new contact within the last 3 months.)

☐☐ 01=Yes, 02=No, 77=Don't remember

3.For how long have you known [Name]? Answer in completed days, weeks, months or years.

☐☐

- ☐ **Days**
- ☐ **Weeks (Probe for estimate)**
- ☐ **Months (Probe for estimate)**
- ☐ **Years(probe for estimate)**

4.For how long has [Name] been your contact? Give answer in completed days, weeks, months or years.

☐☐

- ☐ **Days**
- ☐ **Weeks (Probe for estimate)**
- ☐ **Months (Probe for estimate)**
- ☐ **Years (Probe for estimate)**

5.Did you know [Name] before you started to cough]?

☐☐ 01=Yes, 02=No, 77=Dont remember

6.Since the onset of your cough, has the nature of your relationship with [[Name] changed?

☐☐ 01=Yes, 02=No

7.If "YES" What was the nature of your relationship before the change?

☐☐

01= Spouse
02 = Co-worker
03= Student colleague
04 = Relative
05= Friend
06 = Acquaintance
88= Other (Specify) ☐

8.Since the onset of your cough, would you say the frequency of contact with [Name] has

☐☐

01 = Increased
02 = Decreased
03 = Remained the same

9.How well do you know [Name]?

☐☐

01 = Very well
02 = Moderately well
03 = Somewhat well
04 = Almost do not know him/her
05 = Dont know him/her

10.Do you discuss important life issues with [Name]?

☐☐ 01=Yes, 02=No, 66=No response

11.Do you confide in [Name] with your health concerns?

☐☐ 01=Yes, 02=No, 66=No response

12.Have you shared your TB diagnosis with [Name]?

☐☐ 01=Yes, 02=No

13.Over the past one month, how often do you come into contact with [Name]?

☐☐

01 = None
02 = Less than a day/week
03 = 1-3 days/week
04 = 4-6 days /week
05 = Daily
77 = Dont recall

14.Over the past one month, on the days you had contact with [Name] how much time did you spend together?

☐☐

01 = Just a short time (less than or equal to 1hr/day)
02 = Part of the day (2-6 hrs/day)
03 = Part of the day (7-12 hrs/day)
04 = Most of the day (13-18hrs/day)
05 = Over 18 hrs/day
77 = I don't recall
99 = not applicable

Int Initials:	☐☐	Comp Date:	☐☐ /	☐☐ /	2 0 ☐☐
Rev Initials:	☐☐	Rev Date:	☐☐ /	☐☐ /	2 0 ☐☐
DMO Initials:	☐☐	DMO Date:	☐☐ /	☐☐ /	2 0 ☐☐
CD Initials:	☐☐	CD Date:	☐☐ /	☐☐ /	2 0 ☐☐
			Day	Month	Year

40782

MAKERERE UNIVERSITY- UNIVERSITY OF GEORGIA
COMMUNITY HEALTH AND SOCIAL NETWORKS OF TUBERCULOSIS
SOCIAL NETWORK FORM: TB CASE

Network ID [| | | |]   **Int Date** [|] / [|] / [2|0| |]   **Contact serial#** [|]

15a. Since the onset of your cough, have you shared meals with [Name]? [|] 01=Yes, 02=No
77= Dont remember

15b. If "YES", how frequent? [|]
01 = Less than a day/week
02 = 1-3 days/week
03 = 4-6 days /week
04 = Daily
77 = Dont recall
99 = Not applicable

16a. Since the onset of your cough, have you slept in the same room with [Name]? [|] 01= Yes, 02=No,

16b. If "YES", how frequent? [|]
01 = Less than a day/week
02 = 1-3 days/week
03 = 4-6 days /week
04 = Daily
77 = Dont recall
99 = Not applicable

17a. Since the onset of your cough, have you slept on the same bed with [Name]? [|] 01= Yes, 02=No,

17b. If "YES", how frequent? [|]
01 = Less than a day/week
02 = 1-3 days/week
03 = 4-6 days /week
04 = Daily
77 = Dont recall
99 = Not applicable

18a. Since the onset of your cough, has [Name] provided care for you? [|]
01= Yes,
02=No

18b. If "YES", how frequent? [|]
01 = Less than a day/week
02 = 1-3 days/week
03 = 4-6 days /week
04 = Daily
77 = Dont recall
99 = Not applicable

19. Since the onset of your cough, is there a usual place where you meet [Name]? [|] 01=Yes, 02=No,

If "YES", Ask questions 20-22 OR ELSE skip to 23

20. Where do you usually meet [Name]? [|]
01 = Your home
02 = Friend's home
03 = Relative's home
04 = Work place
05 = School
06 = Worship Center
07 = Club/Association
08 = Bar
09 = Saloon
10 = Gym
11 = Trading Center/Sh[illegible]
12 = In transit(specify) [ ]
88 = Elsewhere(specify) [ ]

21. Since the onset of your cough, how many days per week do you spend with [Name] at this location? [|]
01 = None
02 = Less than a day/week
03 = 1-3 days/week
04 = 4-6 days /week
05 = Daily
77 = Dont recall

22. When you meet with [Name] at this location, how much time do you usually spend with him/her? [|]
01 = Just a short time (less than or equal to 1 hr/day)
02 = Part of the day (2-6 hrs/day)
03 = Part of the day (7-12 hrs/day)
04 = Most of the day (13-18hrs/day)
05 = Over 18 hrs/day
77 = I don't recall

Int Initials:	[|]	Comp Date:	[|] / [|] / [2|0| |]
Rev Initials:	[|]	Rev Date:	[|] / [|] / [2|0| |]
DMO Initials:	[|]	DMO Date:	[|] / [|] / [2|0| |]
CD Initials:	[|]	CD Date:	[|] / [|] / [2|0| |]
			Day / Month / Year

40782

MAKERERE UNIVERSITY - UNIVERSITY OF GEORGIA

COMMUNITY HEALTH AND SOCIAL NETWORKS OF TUBERCULOSIS

SOCIAL NETWORK FORM_TB CASE

Network ID ☐☐☐☐☐

Int Date ☐☐ / ☐☐ / 2 0 ☐☐

Contact serial# ☐☐

23.Where did you **most recently** meet [Name]? ☐☐

01 = Your home
02 = Friend's home
03 = Relative's home
04 = Work place
05 = School
06 = Worship Center
07 = Club/Association
08 = Bar
09 = Saloon
10 = Gym
11 = Trading Center/Sh[illegible]
12 = In transit(specify) ________
88 = Elsewhere(specify) ________

24 .During this meeting, how much time did you spend with [Name]? ☐☐

01 = Just a short time (less than or equal to 1 hr/day)
02 = Part of the day (2-6 hrs/day)
03 = Part of the day (7-12 hrs/day)
04 = Most of the day (13-18hrs/day)
05 = Over 18 hrs/day
77 = I don't recall

25.Do you meet in doors or outdoors? ☐☐

01=Mostly Indoors , 02=Mostly Outdoors
03=Equally inside and outside

26.Reflect on this place where you usually meet. How would you characterize the ventilation of this place? ☐☐

01 = POOR: Completely enclosed (all windows &doors closed)
02 =MINIMAL: Partially closed (some windows and doors closed
03 =FAIR: structure has roof, enclosed in four walls with windows and doors open, typical of a retail shop
04 =FULL: Completely outdoors ,under a tree, under a roof supported by poles
77 =Dont Know

27 .When you meet with [Name] are there other people you know/interact with at this location? ☐☐

01=Yes, 02=No,

If "YES", ask question 28 OR ELSE Skip to 29

28. How many other people? ☐☐

29. Do you meet with [Name] at other locations? ☐☐

01=Yes, 02=No,

If "YES", Ask question 29 OR ELSE skip to 31

30. How often do you meet at other locations? ☐☐

01 = None
02 = Less than a day/week
03 = 1-3 days/week
04 = 4-6 days /week
05 = Daily
77 = Dont recall

31. Since you started to cough, which of the following means of transportation do you share with [Name]?
Cross all that applys

- ☐ **Motor bike**
- ☐ **Train**
- ☐ **Bodaboda**
- ☐ **Plane**
- ☐ **Private vehicle**
- ☐ **Boat**
- ☐ **Taxi**
- ☐ **None**
- ☐ **Lorry**
- ☐ **Bus**
- ☐ **Other** ________

32. Which means of transportation do you use most often with [Name]? ☐☐

01 = Motorcycle
02 = Bodaboda
03 = Private vehicle
04 = Taxi
05 = Lorry
06 = Bus
07 = Train
08 = Plane
09 = Boat
10=None
88= Other ________

33.To your knowledge, does [Name] have cough? ☐☐

01=Yes, 02=No, 77=Dont know

34.To your knowledge, does [Name] have TB? ☐☐

01=Yes, 02=No, 77=Dont know

Int Initials:	☐☐	Comp Date:	☐☐ / ☐☐ / 2 0 ☐☐
Rev Initials:	☐☐	Rev Date:	☐☐ / ☐☐ / 2 0 ☐☐
DMO Initials:	☐☐	DMO Date:	☐☐ / ☐☐ / 2 0 ☐☐
CD Initials:	☐☐	CD Date:	☐☐ / ☐☐ / 2 0 ☐☐
			Day / Month / Year
