# Supplementary material for: Association between tuberculosis in men and social network structure in Kampala, Uganda
Source: BMC Infect Dis. 2021 Sep 30;21:1023. doi: 10.1186/s12879-021-06475-z (PMC8482622; doi:10.1186/s12879-021-06475-z)
Supplement: Supplementary file 2 — Additional file 2. Supplementary analysis. [file 12879_2021_6475_MOESM2_ESM.docx]

Supplementary Information: Association between male-bias in tuberculosis cases and social network structure in Kampala, Uganda

**Glossary of network sampling terms**

Egocentric sampling: Ego-centric network sampling identifies the networks of a set of sampled participants (egos); in this study egos are index individuals

Index individual: Index cases or index controls

Index case: A person who presented to health care for diagnosis and treatment of tuberculosis and who was enrolled in the study

Index control: A person in the community without tuberculosis disease; these individuals were matched to index cases by age, sex, and local parish

First level contact: Listed contacts of index individuals

Second level contact: Listed contacts of first level contacts

**Sensitivity analyses using simulated networks**

To examine potential biases on measures of node position and estimates of assortativity in second-level egocentric sampling, we performed two sensitivity analyses. First, we assessed the reliability of node position estimates from egocentric samples. To account for uncertainty in structure and size of the “true” social network in Kampala, we analyzed two types of networks proposed to resemble real-world social networks (small-world, SW, and scale-free, SF) across five different network sizes $(N=5 \cdot{10}^{4},7.5\cdot{10}^{4},1\cdot{10}^{5},1.25\cdot{10}^{5},1.5\cdot{10}^{5})$. We generated small-world networks with the Watts-Strogatz algorithm [1], starting connectivity of 5, and edge-rewiring probability of 0.05. We generated scale-free networks with the Barabasi-Albert algorithm [2] with linear attachment and 5 edges added in each step. Each combination of network type and size were replicated 15 times and sampled in three different ways: ego-only, first-level, and second-level egocentric sampling. We then calculated the correlation between true node centrality and estimated node centrality for the ego nodes (n=240).

In a second sensitivity analysis on estimates of assortativity in egocentric samples, we generated 15 replicates of synthetic networks across a spectrum of network assortativity (r$\approx0, 0.2, 0.4,0.6, 0.8$) and two different network sizes ($N=1\cdot{10}^{3},1\cdot{10}^{4}$). We generated networks with varying assortativity by first assigning each node to one of two groups (i.e., sex). Then, we set the number of within- and between-group edges per node from Poisson distributions (similar to algorithm in [7]). The mean degree in synthetic networks was 10. For example, in one combination, each node had an average of 5 edges within-group and 5 edges between-group resulting in an overall network assortativity value of approximately 0. Following network simulations, we sampled each network using second-level egocentric sampling type with 240 randomly egos. Finally, we calculated the correlation between sampled assortativity and true assortativity of the underlying network to understand the effects of egocentric sampling on estimates of network assortativity.

**Observed Kampala network fit to using simulated networks**

To understand how the Kampala network relates to simulated networks, we calculated the clustering coefficient (the probability that neighbors of a node are themselves connected [3]) and fit to a power-law degree distribution because small-world networks are characterized by high clustering coefficients and scale-free networks by a power-law degree distributions

[4]. The power law degree distribution was fit to the Kampala data and synthetic networks using the method of maximum likelihood and the goodness-of-fit tests [5]. We additionally evaluated whether other distributions fit the data equally using the Vuong test statistic (approach detailed in [5]; implemented in the R package, poweRlaw [6]).

The degree distribution of the Kampala network could plausibly follow a power-law (Kampala data fell within bootstraps from the best-fit power-law distribution, p=0.25). However, the log-normal distribution could also not be ruled out for the tail of the degree distribution (likelihood-ratio-test showed that neither power-law nor log-normal was preferred, p=0.56). Recommended approach from Clauset et al. (2009) [5].

Tables and Figures


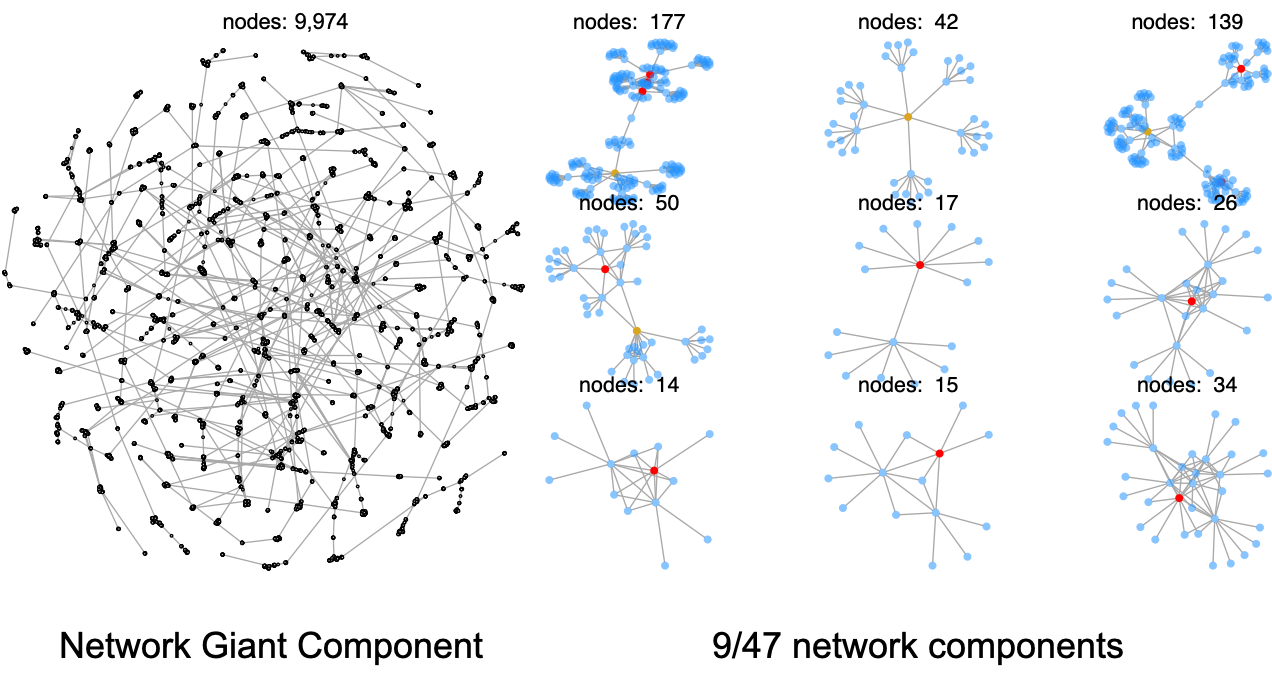


Figure S1. Network giant component (left) and nine of 47 other network components (right). Component size (number of nodes) is shown at the top of each network. Node color represents participant type – red nodes are cases, yellow nodes are controls, blue nodes are first- or second-level contacts.


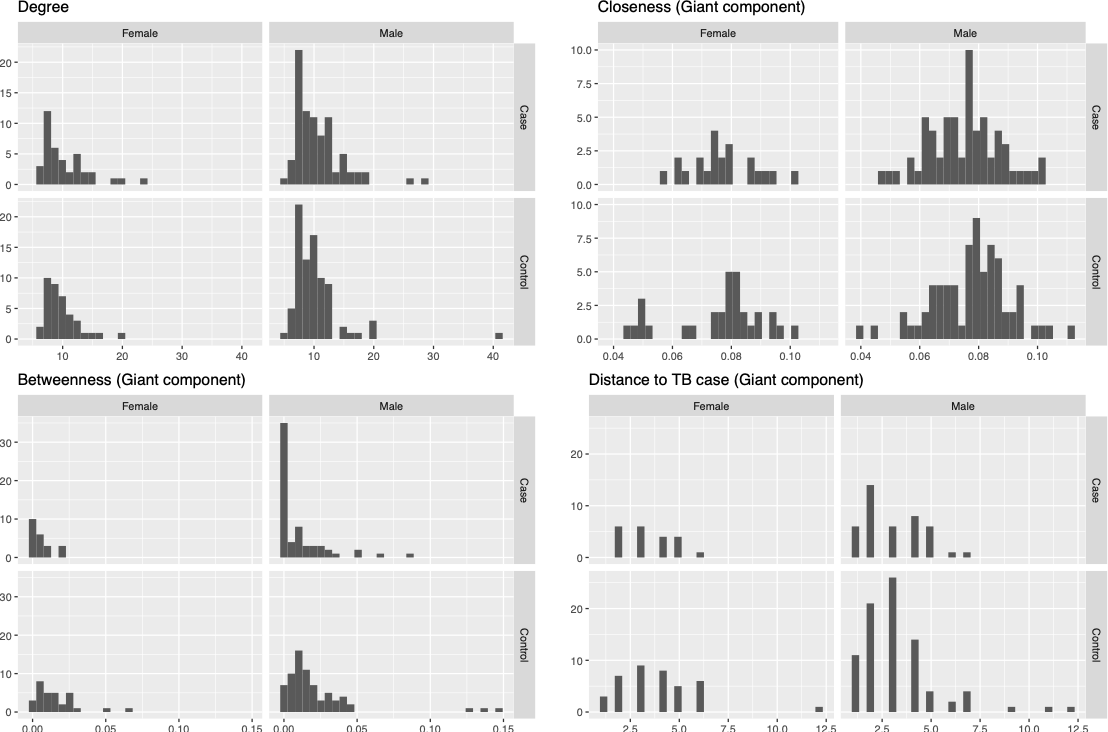


Figure S2. Distributions of node position statistics of index individuals stratified by type (case, control) and sex (male, female). Degree of index individuals was compared for all index individuals while betweenness, closeness, and distance to TB cases were compared only for individuals in the giant component to control for component size and because these statistics are not well-defined for disconnected networks.

Figure S3. Clustering coefficients from simulated networks of varying sizes that were sampled using a second-level egocentric design. We used these distributions to understand whether the underlying network in Kampala more closely resembles scale-free (SF) or small-world (SW) structure. The clustering coefficient of the Kampala network was 0.1, which was in between estimates from scale-free and small-world networks.

Figure S4. The cumulative distribution function of node degree in the Kampala network resembles both power-law and log-normal distributions but not exponential or Poisson distributions. The tail of the distribution was estimated to begin at 13 and values larger than 13 were used to fit the distributions (n=264). A fit to a power-law distribution is an indication of scale-free networks but log-normal could not be ruled out using the methods detailed in Clauset et al. (2009) [5].

Figure S5. Estimated assortativity is highly correlated with underlying network assortativity in second-level egocentric samples from networks of size 1,000 and 10,000 $(\rho>0.99)$. Estimated assortativity in second-level samples tend to underestimate the true amount of assortativity. The dashed grey line indicates the 1-1 line.

**Table S1.** Social Network Estimates for Index Individuals stratified by Relationship Status and Sex. Values indicate the number of individuals (proportion) or mean ($\pm$standard errors) for each variable.

|  | **Single** | | **In a relationship** | |  |
| --- | --- | --- | --- | --- | --- |
|  | **Female**  n=44 | **Male**  n=93 | **Female**  n=34 | **Male**  n=76 | **Sig.** |
| **Node position** | | | | | |
| Degree | $10.6 (\pm0.58)$ | $10.4 (\pm0.41)$ | $9.6 (\pm0.48)$ | $10.8 (\pm0.53)$ |  |
| Closeness | 0.078 $(\pm0.002)$ | $0.075 (\pm0.002)$ | 0.074 $(\pm0.003)$ | $0.078 (\pm0.001)$ |  |
| Betweenness | 0.012 ($\pm0.003)$ | $0.014 (\pm0.003)$ | $0.010 (\pm0.002)$ | $0.019 (\pm0.002)$ |  |
| Distance to case | $3.5 (\pm0.4)$ | $3.2 (\pm0.2)$ | $3.9 (\pm0.3)$ | $3.2 (\pm0.2)$ |  |
| **Mixing variables** | | | | | |
| Proportion of all contacts with adult men | $0.27 (\pm0.03)$ | $0.49 (\pm0.03)$ | $0.29 (\pm0.04)$ | $0.46 (\pm0.03)$ | $*$ |
| Proportion of all contacts with adult women | $0.42 (\pm0.03)$ | $0.38 (\pm0.02)$ | $0.43 (\pm0.04)$ | $0.36 (\pm0.02)$ | $*$ |
| ^1^Proportion of all contacts with children | $0.31 (\pm0.03)$ | $0.13 (\pm0.02)$ | $0.28 (\pm0.03)$ | $0.18 (\pm0.02)$ | $*$ |
| ^2^Proportion of all contacts occurring within HH | $0.33 (\pm0.04)$ | 0.25 $(\pm0.02)$ | 0.33 $\left( \pm0.03 \right)$ | $0.30 (\pm0.02)$ | $*$ |
| Proportion of HH contacts occurring with children | 0.34 $(\pm0.03)$ | 0.28 $(\pm0.02)$ | 0.32 $(\pm0.03)$ | 0.28 $(\pm0.02)$ | $*$ |

$*$ Significant difference (p <0.05 ) between means by index sex (male, female)

$⋄$ Significant difference (p <0.05) between means by relationship status (single, monogamous/polygamous)

REFERENCES**:**

1. Clauset A, Shalizi CR, Newman MEJ. Power-Law Distributions in Empirical Data. SIAM Review. Society for Industrial and Applied Mathematics; 2009;51: 661–703. doi:10.1137/070710111

1. Watts DJ, Strogatz SH. Collective dynamics of “small-world” networks. Nature. Nature Publishing Group; 1998;393: 440–442. doi:10.1038/30918

2. Barabasi A, Albert R. Emergence of scaling in random networks. Science. 1999;286: 509–512.

3. Wasserman S, Faust K. Social network analysis: Methods and Applications. Cambridge University Press. 1994;8.

4. Kiss IZ, Miller JC, Springer PSC. Mathematics of epidemics on networks. Springer

. 2017.

5. Clauset A, Shalizi CR, Newman MEJ. Power-Law Distributions in Empirical Data. SIAM Review. Society for Industrial and Applied Mathematics; 2009;51: 661–703. doi:10.1137/070710111

6. Gillespie CS. Fitting Heavy Tailed Distributions: The poweRlawPackage. Journal of Statistical Software. 2015;64: 1–16. doi:10.18637/jss.v064.i02

7. Girvan M, Newman MEJ. Community structure in social and biological networks. PNAS. National Academy of Sciences; 2002;99: 7821–7826. doi:10.1073/pnas.122653799
